# Improved Long-Term Survival of Patients with Recurrent Medulloblastoma Treated with a “MEMMAT-like” Metronomic Antiangiogenic Approach

**DOI:** 10.3390/cancers14205128

**Published:** 2022-10-19

**Authors:** Irene Slavc, Lisa Mayr, Natalia Stepien, Johannes Gojo, Maria Aliotti Lippolis, Amedeo A. Azizi, Monika Chocholous, Alicia Baumgartner, Cora S. Hedrich, Stefan Holm, Astrid Sehested, Pierre Leblond, Karin Dieckmann, Christine Haberler, Thomas Czech, Marcel Kool, Andreas Peyrl

**Affiliations:** 1 Department of Pediatrics and Adolescent Medicine, Medical University of Vienna, Waehringer Guertel 18-20, 1090 Vienna, Austria; 2 Comprehensive Center for Pediatrics, Medical University of Vienna, 1090 Vienna, Austria; 3 Department of Women’s and Children’s Health, Karolinska Institutet, 17177 Stockholm, Sweden; 4 Astrid Lindgrens Children’s Hospital, Karolinska University Hospital, 17164 Stockholm, Sweden; 5 Department of Paediatrics and Adolescent Medicine, Copenhagen University Hospital Rigshospitalet, 2100 Copenhagen, Denmark; 6 Institute of Pediatric Hematology and Oncology IHOPe, Léon Bérard Cancer Center, 69002 Lyon, France; 7 Department of Pediatric Oncology, Oscar Lambret Cancer Center, 59000 Lille, France; 8 Radiation Oncology, Department of Radiotherapy, Medical University Vienna, 1090 Vienna, Austria; 9 Division of Neuropathology and Neurochemistry, Department of Neurology, Medical University of Vienna, 1090 Vienna, Austria; 10 Department of Neurosurgery, Medical University of Vienna, 1090 Vienna, Austria; 11 Hopp Children’s Cancer Center (KiTZ), 69120 Heidelberg, Germany; 12 Division of Pediatric Neurooncology, German Cancer Research Center (DKFZ), German Cancer Consortium (DKTK), 69120 Heidelberg, Germany; 13 Princess Máxima Center for Pediatric Oncology, 3584 Utrecht, The Netherlands

**Keywords:** medulloblastoma recurrence, antiangiogenic therapy, metronomic therapy, low-dose oral therapy, bevacizumab, intraventricular therapy, MEMMAT

## Abstract

**Simple Summary:**

About 30% of patients with medulloblastoma experience recurrence, which is usually incurable despite intensive chemotherapy. The aim of our retrospective study was to evaluate a novel combinatorial metronomic antiangiogenic approach (“MEMMAT-like”) for recurrent medulloblastoma consisting of five oral drugs, an intravenous antibody against vascular endothelial growth factor, and intrathecal therapy. The study, conducted between 2006 and 2016, included 29 consecutive patients with first or multiple recurrences treated according to this “MEMMAT-like” strategy and confirmed a significantly longer median overall survival than in previously reported studies. As of 07/2022, 9/29 patients are alive 86 to 164 months after recurrence. Treatment was primarily out-patient and well-tolerated. Toxicities did occur but were manageable. The novel combination significantly improved overall and progression-free survival for patients with recurrent medulloblastoma. A formal study (MEMMAT; ClinicalTrials.gov Identifier: NCT01356290) has been completed and is currently being evaluated.

**Abstract:**

Medulloblastoma (MB) recurrence is usually incurable despite intensive therapy including high-dose chemotherapy. An evolving alternative approach to conventional chemotherapy aims at interfering with tumor angiogenesis at different levels. We report on a novel combinatorial metronomic antiangiogenic approach. The study is a retrospective observational study of 29 consecutive patients with first or multiple recurrences prospectively treated according to the MEMMAT strategy (“MEMMAT-like”) before the formal protocol (MEMMAT; ClinicalTrials.gov Identifier: NCT01356290) started. The study period was 11/2006 to 06/2016. Treatment consisted of daily oral thalidomide, fenofibrate, celecoxib, and alternating 21-day cycles of low-dose oral etoposide and cyclophosphamide supplemented by IV bevacizumab and intraventricular therapy consisting of alternating etoposide and liposomal cytarabine. Median overall survival (OS) after recurrence for the whole group was 29.5 months, OS was 48.3 ± 9.3% at three years and 34.5 ± 8.8% at five years, and progression-free survival was 42.0 ± 9.5% at three years and 29.4 ± 9% at five years. As of 07/2022, 9/29 patients are alive 86 to 164 months after the recurrence that prompted the “MEMMAT-like” therapy. Treatment was primarily out-patient and generally well-tolerated. Toxicities did occur but were manageable. In conclusion, antiangiogenic therapy according to the MEMMAT strategy increased median OS of patients with recurrent MB and may lead to long-term survival. Adherence to the protocol, including intraventricular therapy, appears important.

## 1. Introduction

Medulloblastoma (MB), an embryonal neoplasm arising in the cerebellum or dorsal brain stem, is one of the most common malignant CNS tumors, accounting for up to 10% of all childhood CNS tumors [1]. Since the 1970s, a variety of factors, including advances in neuroimaging, neurosurgical techniques, neuroanesthesiology, radiation, and chemotherapy, led to a steady increase in survival of newly diagnosed MB patients. Over the past decade, however, the improvement in survival has leveled off at a 5-year survival rate of 65–80% [2,3]. The best outcome is achieved in patients without metastases who underwent a gross total or near total surgical resection of their tumor and who received craniospinal irradiation and adjuvant chemotherapy [4,5]. Recently, methylome and transcriptome profiling of MBs revealed that MB is a heterogeneous tumor comprising various groups with distinct developmental origins, transcriptional profiles, and diverse phenotypes, all of which reflected in clinical outcome [6,7,8,9,10]. The 2016 update of the World Health Organization (WHO) classification of CNS tumors recognized four molecularly distinct groups: Wingless (WNT)-activated, Sonic Hedgehog (SHH)-activated, and Group 3 and Group 4 MB [11]. In contrast to WNT and SHH MBs that exhibit specific alterations in the respective signaling pathways, no specific oncogenic pathways were identified for Group 3 and 4 MBs, and all molecular groups have already been shown to encompass further subgroups [12,13,14,15]. These subgroups, some of which may also provide clinical utility, have now also been incorporated in the most-recent and fifth edition of the WHO classification of CNS tumors [16]. 

In general, WNT MB patients younger than 16 years at diagnosis carry the best prognosis, with 5-year overall survival rates of more than 90%. [17,18] In contrast, *MYC-*amplified Group 3 MBs have the worst outcome of all subgroups, whereas SHH and Group 4 MBs show an intermediate prognosis [19,20,21]. 

Despite these advances, approximately 30% of MB patients recur and prognosis following MB relapse is extremely poor, with recent series reporting an overall survival of only around 20% at 3 years and 6 to 12% at 5 years [22,23,24,25,26]. Phase 1/2 trials studying the efficacy of a single novel agent for relapsed MB usually failed to improve long-term survival, and there is no standard therapy for these highly aggressive tumors [27,28,29,30]. 

An alternative approach to conventional chemotherapy or single agent targeted therapy is a metronomic chemotherapy (MC) [31,32]. MC is defined as long-term administration of chemotherapeutic agents at relatively low, minimally toxic doses and with no prolonged drug-free breaks [33]. Browder et al. demonstrated that cyclophosphamide-resistant tumors can be killed in vivo by metronomic dosing of the same drug, and that this dosing schedule inhibits tumor growth primarily through antiangiogenic mechanisms [34]. This observation has been replicated with other cytotoxic drugs [35,36,37,38]. Furthermore, the term “metronomics” is not restricted to chemotherapeutic agents but may comprise repositioning or repurposing of nonchemotherapeutic drugs as well [33]. Because of the redundancy of mechanisms involved in the formation of new blood vessels by cancer growth, induction and maintenance of tumor response requires interfering with multiple pathways [39]. 

An early feasibility trial that tested such a combinatorial metronomic antiangiogenic approach in twenty consecutive children with various recurrent/progressive cancers was published by Kieran et al. in 2005 [40]. The “4-drug” regimen consisted of alternating 21-day cycles of low-dose oral cyclophosphamide and etoposide, with continuous oral thalidomide and celecoxib using antiangiogenic doses for all four drugs. Based on subsequent preclinical experiments demonstrating an antiangiogenic and antitumor activity for fenofibrate, a PPAR-alpha agonist, as well as a synergistic effect of combining metronomic etoposide with PPAR modulation and COX-2 inhibition, the consecutive phase II “5-drug” regimen included fenofibrate into the metronomic armamentarium [41,42]. 

Prompted by an MB patient with a second recurrence, who was treated on the “5-drug” trial and achieved an impressive, almost-complete response but recurred three months after discontinuation of metronomic treatment, we decided to restart the “5-drug” regimen in this patient. It was augmented with bevacizumab, a humanized monoclonal antibody binding all five isoforms of human vascular endothelial growth factor (VEGF), and intraventricular therapy via an Ommaya reservoir [41]. This approach evolved into an international phase II trial (MEMMAT; ClinicalTrials.gov Identifier: NCT01356290). Here, we report on the survival of 29 consecutive patients with recurrent MB treated at four pediatric cancer centers in Europe before the formal trial started. Potential associations between survival and molecular subgroup were evaluated as a secondary objective.

## 2. Patients and Methods

The study is a retrospective observational study of 29 consecutive patients diagnosed with a recurrent MB in four different centers across Europe (Vienna, Stockholm, Copenhagen, and Lille). Patients were prospectively treated per/off protocol according to the MEMMAT strategy (henceforth referred to as “MEMMAT-like”) before the formal protocol (MEMMAT; ClinicalTrials.gov Identifier: NCT01356290) started or parallel to it if they met exclusion criteria of the formal protocol such as a VP shunt in place. The study period was 11/2006 to 06/2016. Preliminary results of seven patients were included in an early case series with this antiangiogenic approach in various embryonal brain tumors [43]. Data were collected by the local investigators and submitted anonymously for final analysis. Information on date of primary diagnosis, age, gender, metastatic stage, pathology based on histologic classification, molecular data if available, treatment received at diagnosis, date and number of prior relapses, date of relapse that prompted “MEMMAT-like” treatment, adverse effects associated with “MEMMAT-like” treatment, treatment after “MEMMAT-like”, and clinical outcome was collected. The survey protocol and retrospective analysis of the data was approved by the local ethics board of the coordinating institution and additionally by institutional review boards, as required. (EK Nr: 2041/2020)

### 2.1. Prior Treatment

Primary treatment followed the MB protocols in use in the respective countries at the time, which was SIOP PNET4 in nine [44], HIT 2000 in nine [45], HIT 91 in three [4,46], “Head start” III in one [47], COG A9961 in one [48], other in five, and radiotherapy only in one (case 16). Tumor stage at primary diagnosis was M0 in 14, metastasized in 9 patients, and suspicious for tumor cells in the cerebrospinal fluid (CSF) (depicted as M0-1) or not known in the remaining (Table 1). Male-to-female ratio was 19:10. Except for three patients who were below one-year of age at diagnosis, all patients were irradiated at primary diagnosis. Five patients were reirradiated for prior relapses. In accordance with the guidelines of the participating institutions, informed consent was obtained from the parents or legal guardians of the patients.

### 2.2. Histopathology

Tumor tissue from primary diagnosis was reviewed by the respective study pathologists confirming the diagnosis and the histomorphological type. Depending on the trial protocol (HIT SIOP PNET 4 and HIT 2000), additional MB biomarkers were investigated as part of the study. Immunohistochemistry (IHC) for β-catenin, YAP1, GAB1, and TP53 was performed to define molecular subgroup in 17 of 29 patients. Additionally, sequencing of exon 3 of *CTNNB1* was carried out to confirm the diagnosis of a WNT-subgroup MB. 

### 2.3. Tumor Molecular Profiling

Tumor specimens were analyzed using 450K or 850K BeadChip arrays (Illumina, San Diego, CA, USA) from either freshly frozen or FFPE tissue. MB Group and subgroup predictions were determined using DNA methylation-based classification of CNS tumors (www.MolecularNeuropathology.org, accessed on 20 September 2022; Heidelberg, Germany, version 12.5) at the German Cancer Research Center (DKFZ) in Heidelberg, as previously described [49]. Calibration scores of >0.9 were used as cutoff for classifying these tumors as MB and for (sub)group classification using the classifier. For tumors with calibration scores < 0.9 but still predicted to be MB, t-distributed stochastic neighbor embedding (tsne) clustering analysis was performed to classify them into different (sub)groups. Genome-wide DNA copy number alterations were inferred from DNA methylation arrays using the Conumee R package [50].

### 2.4. Patient Evaluation

A full medical history was obtained before the start of “MEMMAT-like” antiangiogenic treatment. All patients underwent physical (including blood pressure) and neurological examination, performance status evaluation, routine laboratory tests including blood chemistry and urine analysis, and MRI scans. A lumbar puncture for CSF evaluation regarding tumor cells was recommended if safe, alternatively ventricular CSF obtained via the Ommaya reservoir could be used. MRI scans were repeated at least every three months during treatment and clinical and laboratory tests at least every other week. Toxicity was evaluated according to the National Cancer Institute (NCI) Common Terminology Criteria for Adverse Events (CTCAE) version 4.0 criteria.

### 2.5. “MEMMAT-like” Antiangiogenic Treatment

**Figure 1:**
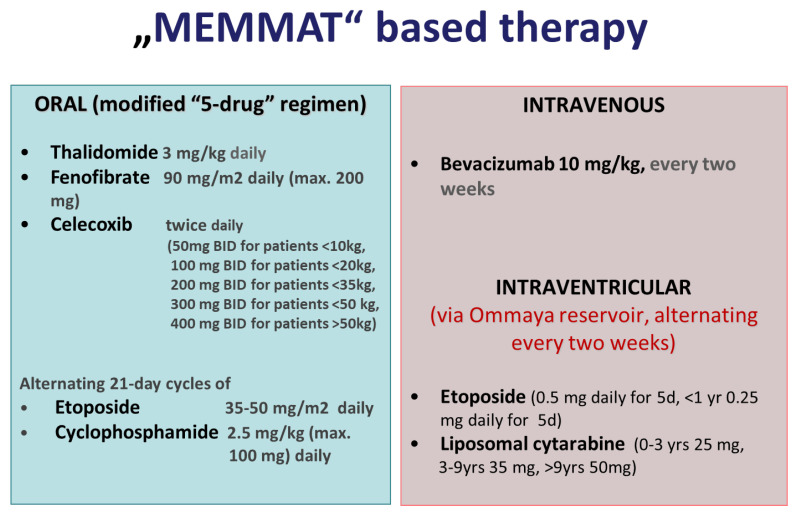
Drugs, dosing, and schedule of MEMMAT-based therapy.

Treatment consisted of a modified “5-drug” oral regimen including daily oral thalidomide, daily oral fenofibrate, twice daily oral celecoxib, and alternating 21-day cycles of low-dose oral etoposide and cyclophosphamide [42] as a backbone supplemented by IV bevacizumab every two weeks and intraventricular therapy consisting of alternating etoposide and liposomal cytarabine. (Figure 1). In contrast to the original “5-drug” regimen that suggested a weekly increase in thalidomide by 50 mg to a total dose of 24 mg/kg (max. 1000 mg), thalidomide was initiated at the same dose of 3 mg/kg but not increased, and rather lowered, in case of side effects such as numbness, tingling, and decreased nerve conduction velocity. Planned treatment duration was one year. In case of response, continuation of treatment was recommended for a second year without oral etoposide and cyclophosphamide and with extended intervals of intraventricular therapy. Dose reductions were recommended to best avoid interruption of treatment due to toxicity at the discretion of the treating institution’s physician. Additional radiotherapy to focal residues concomitant to “MEMMAT-like” therapy was permitted and recommended after response evaluation by MRI.

### 2.6. Intraventricular Therapy

Intraventricular therapy consisted of etoposide (0.25 mg patients < 1 year of age; 0.5 mg > 1 year of age) on five consecutive days [51,52,53] alternating with liposomal cytarabine (DepoCyte ^®^) (<3 years 25 mg, >3 and <9 years 35 mg, and 35–50 mg for children >9 years in combination with oral dexamethason to prevent chemical meningitis) every two weeks [54,55]. Shortly after completion of this cohort, production of liposomal cytarbine (DepoCyte) was discontinued, and liposomal cytarabine was substituted by aqueous cytarabine 30 mg twice a week on days 1, 4, 8, and 11 (<1 year 16 mg, >1 and <2 years 20 mg, >2 and <3 years 26 mg). To avoid potentially toxic effects related to benzyl alcohol, ETO-GRY^®^ (Teva, Ulm, Germany) was used [51], which contained etoposide and ethanol, macrogol 300, polysorbate 80, and citric acid. 

### 2.7. Treatment Response and Toxicity Evaluation

Gadolinium-enhanced and -nonenhanced MRIs were performed as per institutional guidelines. Response was assessed by MRI performed at enrollment and every three months thereafter until tumor progression. Either T1- or T2-weighted images of all target lesions—whichever gave the best estimate of tumor size as determined by the treating team—were used for the measurements of the longest tumor dimension and its perpendicular. The overall response assessment took into account response in both target and nontarget lesion and the appearance of new lesions. Best response was regarded as best response at any single assessment. A complete response (CR) was defined as complete disappearance of measurable disease by MRI, a partial response (PR) as ≥50% decrease in the product of the two maximum perpendicular diameters relative to the baseline evaluation, stable disease as ≤50% decrease and ≤25% increase in product of diameters, and progressive disease (PD) as ≥25% increase in product of diameters or development of new areas of disease according to the criteria for Response Assessment in Pediatric Neuro-Oncology (RAPNO) [56]. Side effects were retrospectively collected and categorized according to the Common Terminology Criteria for Adverse Events (CTCAE) v.4. 

### 2.8. Statistical Methods

Progression-free survival (PFS) was defined as time from diagnosis of recurrence that prompted “MEMMAT-like” treatment to date of relapse or progressive disease. Event-free survival (EFS) was defined as time from diagnosis of recurrence that prompted “MEMMAT-like” treatment to date of relapse or progressive disease or death from any cause, or to date of last follow-up for patients without events. Overall survival (OS) was defined as time from date of relapse that prompted “MEMMAT-like” treatment to date of death from any cause or to date of last follow up for survivors. Kaplan–Meier survival estimates were used in the analysis of overall survival, progression-free survival, and event-free survival rates. Molecular groups were compared for significant differences using the log-rank test. Level of statistical significance was 95%. Statistical analysis was performed using the software package IBM SSPS Statistics version 28.

## 3. Results

### 3.1. Diagnosis of Recurrence

Twenty-seven relapses were detected on surveillance MRI scans, and two patients (cases 5 and 16) presented with symptoms. Except for four patients, who received different types of chemotherapy to bridge the time from diagnosis of relapse to start of “MEMMAT-like” treatment (cases 5, 11, 12, and 16), all patients had relapsed/progressive disease when entering “MEMMAT-like” treatment. Of the four patients who had received multiagent chemotherapy before starting “MEMMAT-like” therapy, three had SD (5, 11, and 12) and one (case 16) a PR at time of enrollment in “MEMMAT-like” treatment.

Nineteen patients had first recurrences and ten had multiple recurrences. Twenty-two patients had metastatic recurrences, three had combined recurrences, and four had local recurrences only. Tissue confirmation for the current or a prior relapse to exclude second malignancy was available in 11 patients. Six patients had positive CSF cytology in addition to manifest leptomeningeal metastases (M1–M3) (Table 1). Median time elapsed from primary diagnosis or prior relapse (in case of multiple recurrences) to relapse that prompted start of “MEMMAT-like” treatment was 21 months (range 3 months to 11 years) (Table 1).

### 3.2. Patients with a Ventriculoperitoneal (VP) Shunt in Place

Eight patients had a VP shunt in place and one had a subduroperitoneal shunt (case 19). In case of a VP shunt, the Ommaya reservoir was placed on the contralateral side and an on-off device (on-off Flushing Reservoir, Integra NeuroSciences, Plainsboro, NJ, USA) inserted into the shunt tubing to enable reversible occlusion of the shunt [57]. Median age (rounded up and down to the nearest whole number) at start of antiangiogenic therapy was 10 years (range 1–24) (Table 1).

### 3.3. Histopathology 

Histopathology was classic MB in 23, large cell anaplastic (LCA) in 3, desmoplastic/nodular in 2 patients, and not available in 1. MB was confirmed histologically at first or subsequent recurrence by surgery in nine patients (cases 1, 5, 6, 9, 10, 11, 13, 16, and 18), by autopsy in two (cases 14 and 20), and by CSF cytology in six (cases 3,12,17, 24, 25, and 28). Nuclear ß-catenin IHC status was known in 21 patients and *CTNNB1* mutation status confirmed in 1/29. None of the 17 patients for whom p53 immunostaining results were available stained positive for p53.

### 3.4. Response and Clinical Outcome after Relapse That Prompted “MEMMAT-like” Treatment

**Figure 2:**
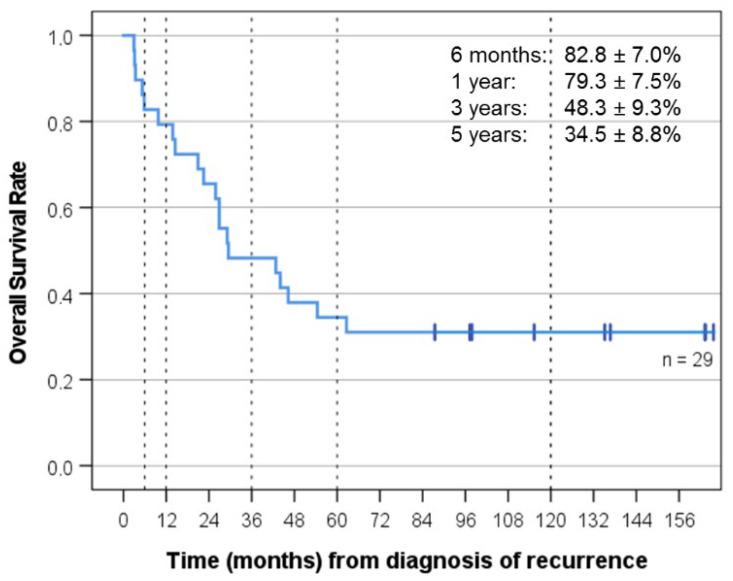
Overall survival (OS) for all 29 patients from time of diagnosis of recurrence that prompted MEMMAT-like therapy. Median OS was 29.5 months (KI 2-57).

**Figure 3:**
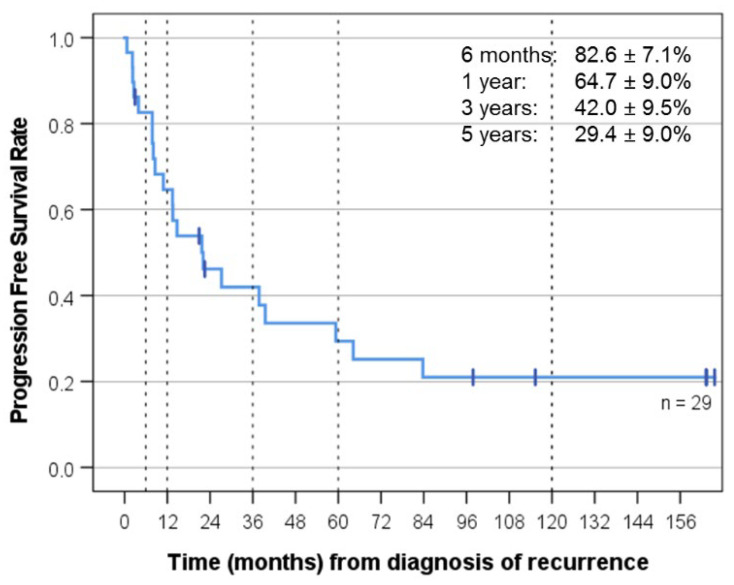
Progression-free survival (PFS) for all 29 patients from time of diagnosis of recurrence that prompted MEMMAT-like therapy. Median PFS was 22.1 months (KI 6-39).

**Figure 4:**
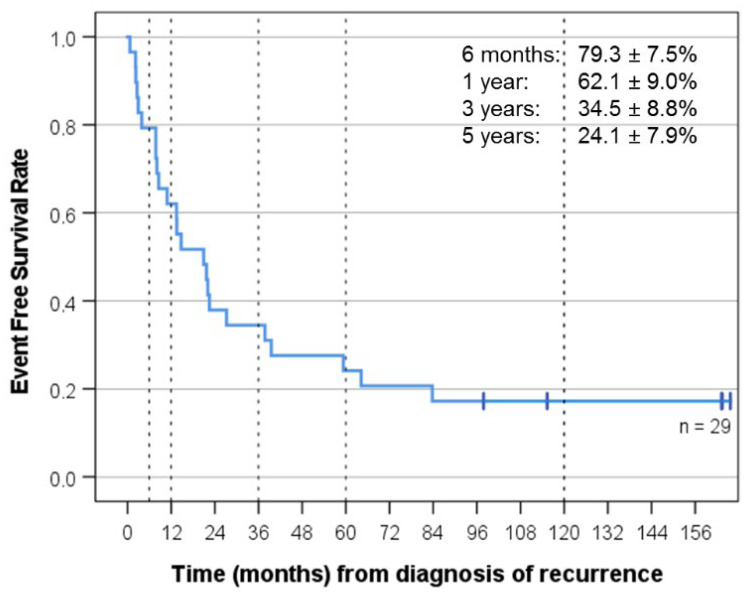
Event-free survival (EFS) for all 29 patients from time of diagnosis of recurrence that prompted MEMMAT-like therapy. Median EFS was 21.0 months (KI 7-35).

Best response was CR (11), PR (8), SD (5), PD (4), and not evaluable because of gross total resection of the recurrence (1). Median follow-up for the whole group was 29.5 months, and median follow-up of the surviving patients was 135.2 months (KI 77-139). OS was 48.3 ± 9.3% at three years and 34.5 ± 8.8% at five years, and PFS was 42.0 ± 9.5% at three years and 29.4 ± 9% at five years. EFS was 34.5 ± 8.8% at three years and 24.1 ± 7.9% at five years (Figure 2, Figure 3 and Figure 4). As of 07/2022, 9/29 patients are alive 86 to 164 months after recurrence that prompted “MEMMAT-like” therapy and 5/9 surviving patients (cases 1, 2, 3, 8, and 23) are currently in continuous complete remission (CCR) between 96 and 164 months after recurrence that prompted enrollment in “MEMMAT-like” therapy (Table 1). 

### 3.5. Molecular Profiling and Outcome Depending on Group Allocation

MB molecular (sub)group classification was determined by DNA methylation array (brain classifier mnp_v12.5) in 23 patients, was non-WNT/non-SHH by immunohistochemistry in 2 additional patients, and not available in the remaining patients (Table 1). 

**Figure 5:**
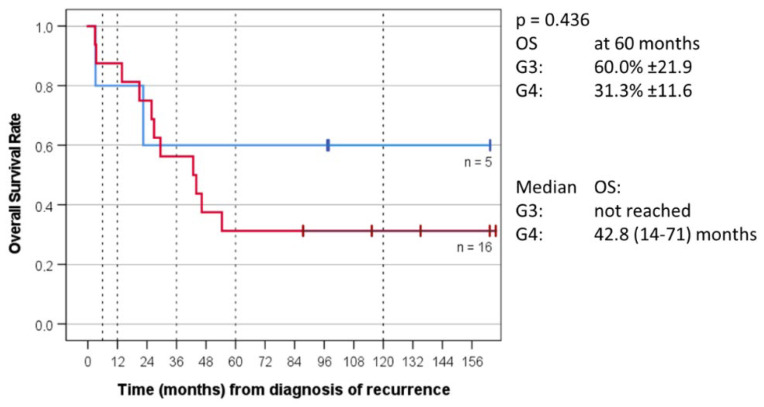
Overall survival (OS) of MB_G3 versus MB_G4 of 21 patients with recurrent non-WNT/non-SHH medulloblastoma for which molecular group was known.

**Figure 6:**
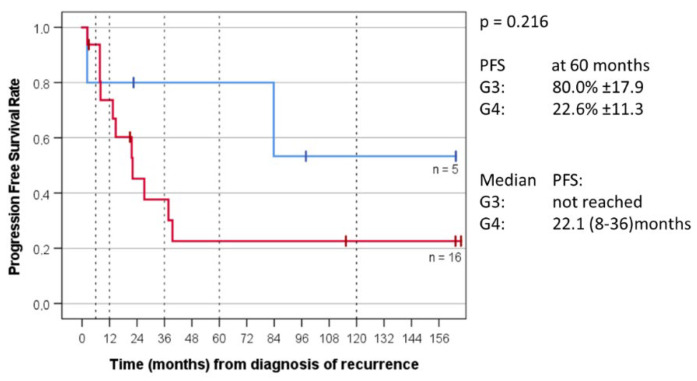
Event-free survival (EFS) of MB_G3 versus MB_G4 of 21 patients with recurrent non-WNT/ non-SHH medulloblastoma for which molecular group was known.

For the 21 non-WNT/non-SHH group patients for whom the MB molecular group was determined, OS at five years was 60.0% ± 21.9 for MB Group 3 and 31.3% ± 11.6 for MB Group 4. Median OS for Group 3 was not reached and was 42.8 months (14–71) for Group 4. PFS at five years was 80.0% ± 17.9 for MB Group 3 and 22.6% ± 11.3 for Group 4. Median PFS for Group 3 was not reached and was 22.1 months (8–36) for Group 4 (Figure 5 and Figure 6).

Three of our long-term survivors had Group 3 tumors (cases 2, 8, and 29) and one patient with subgroup G34_V was classified as Group 3 by methylation array at index diagnosis and Group 4 at recurrence (case 9), four had Group 4 tumors (cases 1, 3, 13, and 23), and for one surviving patient molecular group was not available (case 5). Group and subgroups are listed in Table 1. Clinical details of selected patient groups are summarized in Supplementary Materials.

### 3.6. Treatment after “MEMMAT-like”

Treatment in case of recurrence after discontinuation of “MEMMAT-like” treatment was again “MEMMAT-like” treatment for seven patients (cases 4, 5, 9, 10, 11, 13, and 21) followed by everolimus in one of those (case 9). Four patients received TEMIRI (cases 22, 24, 26, and 27) [58] and one local radiotherapy to the spinal metastasis followed by temozolomide (TMZ) 13 months later (case 29).

### 3.7. Feasibility and Tolerability of “MEMMAT-like” Treatment

Treatment was generally well-tolerated and out-patient in the majority of patients. Sixteen patients completed one year of “MEMMAT-like” treatment and five completed at least 9–11 months of treatment. Depending on number of recurrences, time elapsed from prior treatment, and, thus, bone marrow tolerance, the starting dose of oral etoposide and cyclophosphamide was lowered after a median of 28 days (range 4–166 days) to allow for continuous treatment and avoid interruptions. Median percent of the recommended dose of cyclophosphamide received during the first year of “MEMMAT-like” treatment was 63.5% of the recommended dose (range 34–100%), and median percent of the recommended etoposide dose was 62% (range 39–100%). Thalidomide was lowered to 2.5 mg or 2 mg if necessary. All except for two patients with local recurrences only and a VP shunt in place received intraventricular therapy.

### 3.8. Toxicity

The most common toxicities were grade 3–4 hematologic toxicities requiring reduction and temporary interruptions of one or more drugs. Except for five patients, all patients experienced at least one infectious episode, as evidenced by increase in the C-Reactive Protein (CRP), albeit often without fever and other symptoms due to the COX-inhibition by celecoxib. One heavily pretreated patient died of a septicemia (case 20). Two patients developed a perianal and perigastrostomy fistula, respectively (cases 25 and 27). One patient developed Pneumocystis Jiroveci pneumonia (case 7). Two patients required exchange of their Ommaya reservoir because of infection and cyst formation, respectively (cases 24 and 28). One long-term survivor (case 29) developed severe pneumonitis with acute respiratory distress syndrome requiring mechanical ventilation 12 months after starting “MEMMAT-like” therapy. Bevacizumab induced high blood pressure, requiring antihypertensive medication occurred in four patients (cases 9, 11, 12, and 22) and proteinuria grade 2 also in four patients (cases 6, 9, 22, and 29). Cyclophosphamide induced hematuria grade 1 occurred in three patients (cases 5,10, 14, 23, and 26) and was treated with temporary interruption of medication in all patients and additional uromitexan (mesna) in two (cases 14 and 26). Five patients developed a secondary leukemia (one AML-M3, one AML-MLL, one AML with RUNX1–CBFA2T3 fusion, one MDS-AML with CEPBA double mutation in blasts, one AML NOS). Except for the patient with the MDS-AML and a constitutional karyotype: 46,XY,del(11) (p12p14) [5]/46,XY [15] with CEPBA double mutation in blasts who was only irradiated for primary treatment, all were heavily pretreated for primary metastatic disease and/or prior relapses. Two of these patients are alive and currently in remission of their leukemia and medulloblastoma (case 13 with AML with RUNX1–CBFA2T3 fusion and case 9 with AML-M3). One (case 10 with AML-MLL) died in remission of his medulloblastoma of septicemia during bone marrow transplant, and two were not in remission of their brain tumor (cases 16 and 27) and thus not eligible for bone marrow transplant.

Intraventricular therapy was generally well-tolerated. In case of rare side effects such as headache, fatigue, double vision, vomiting, or seizures, the next dose was delayed, and intraventricular treatment resumed after disappearance of the side effects. Liposomal cytarabine only was discontinued early in four patients because of headaches or perceived fatigue interfering with school performance (cases 9, 22, 23, and 24).

## 4. Discussion

MB relapse has a dismal prognosis with few long-term survivors reported in the literature despite intensive therapy including re-resection, reirradiation, high dose chemotherapy followed by peripheral stem cell rescue, or enrollment in targeted therapy trials. Here, we report on the long-term follow-up of 29 patients with MB recurrences treated with a novel combinatorial antiangiogenic metronomic therapy that demonstrated median EFS and OS superior to previously published series [24,25,29]. In contrast to a conventional cytotoxic chemotherapy given in a dose-intensive fashion to maximize tumor cell kill, a low-dose metronomic combinatorial approach aims at inhibiting multiple angiogenic pathways targeting nonoverlapping aspects of neovascularization. However, an optimal metronomic antiangiogenic approach with regard to drug combination, scheduling, and dosing for a particular tumor type has yet to be defined [36,59,60,61]. The used “MEMMAT-like” combination was based on the 5-drug regimen enhanced by bevacizumab and augmented by intraventricular therapy. The rationale for combining fenofibrate, celecoxib, thalidomide, and low-dose etoposide and cyclophosphamide has previously been described [40,41,42,62]. The addition of the monoclonal antibody bevacizumab was novel in 2006 when the index patient resumed antiangiogenic treatment for his third recurrence and was aimed at blocking VEGF as mediator of tumor angiogenesis. Bevacizumab acts by selectively binding circulating VEGF, thereby inhibiting the binding of VEGF to its cell-surface receptors. Interestingly, this first patient demonstrated a minor response even to bevacizumab monotherapy lasting for 17 months, as demonstrated by MRI. While single case reports, small case series, and early phase trials hinted at an effect of bevacizumab in MB [63,64,65], its role in the treatment of recurrent MB was only recently confirmed in a randomized study. The COG phase II screening study randomly assigned patients with relapsed/refractory MB to receive TMZ and irinotecan with or without bevacizumab [66]. Median OS of the 85 MB patients treated on study was 11 months for the standard arm and 19 months with the addition of bevacizumab, and the median EFS was 5 months in the standard arm and 10 months with the addition of bevacizumab. The authors concluded that the addition of bevacizumab to TMZ/irinotecan significantly reduced the risk of death in children with recurrent MB. Median OS and EFS in our 29 patients treated according to the “MEMMAT-like” approach was 29.5 months and 21 months, respectively, and thus was significantly longer. While the addition of bevacizumab to the 5-drug regimen versus TMZ/irinotecan might have been decisive for the longer median OS and EFS, it is also possible that the addition of intraventricular therapy contributed to the difference. In order to cure an embryonal brain tumor with a high propensity for leptomeningeal dissemination with chemotherapy alone, cytocidal drug levels have to be achieved in the brain tumor itself, in the brain parenchyma, and in the CSF. For most drugs, the CSF drug levels after a systemic dose are less than 20% of the systemic levels because of the problems posed by the blood/brain/CSF barrier [67]. Likewise, antiangiogenic therapy does not reach tumor cells floating in the CSF.

Intrathecal methotrexate in combination with systemic chemotherapy has documented antitumor activity and helped to avoid radiotherapy in newly diagnosed gross-totally resected infant medulloblastomas [68]. Furthermore, Du et al. described 60 children with relapsed MB who were treated with 11 cycles of intensive IV chemotherapy followed by 12 cycles of oral TMZ and etoposide [69]. Half of them received additional, simultaneous intrathecal methotrexate. Interestingly, patients who received intrathecal methotrexate seemed to have benefitted and showed a significantly longer survival than those who did not, supporting the importance of treating the CSF. However, intrathecal methotrexate carries a risk of leukencephalopathy, particularly after prior radiotherapy [70,71]. Given our long-standing experience with various drugs suitable for intrathecal therapy, we decided to use intraventricular etoposide alternating with liposomal cytarabine (DepoCyte) administered via an Ommaya reservoir in all patients with recurrent MB treated according to the MEMMAT approach [52,54,55]. Consequently, all except for two early patients, case 6 with a WNT group tumor and case 14 with a LCA tumor for whom MB molecular group was not available, both with local recurrences only and a VP shunt in place, did not received intraventricular therapy. Unfortunately, both patients who did not receive intraventricular therapy developed combined local and distant recurrences and succumbed to their disease. The potential importance of intrathecal therapy also for WNT pathway tumors is supported by a study by Korshunov, who reported on 78 patients treated for primary WNT MBs at the Burdenko Neurosurgical Institute. Remarkably, 12 of those patients with an advanced stage M2-3 disease at diagnosis had an excellent outcome. Treatment in those 12 patients consisted of two cycles of HIT SKK protocol, which requires intraventricular methotrexate, followed by standard HIT protocol, suggesting a benefit of this approach for advanced stage disease [72].

Intraventricular therapy was generally well-tolerated, and no patient developed a leukoencephalopathy as described for methotrexate despite prior craniospinal irradiation in all except three patients and additional reirradiation for prior or the current relapse in the majority of patients.

Regarding the role of reirradiation, the MEMMAT strategy allows the addition of concomitant focal irradiation after six months of treatment and recommends it in cases of residual tumor, if possible. Recently, several authors retrospectively assessed the role of reirradiation in recurrent/progressive MB in highly selected cohorts of patients and reported a trend toward a better survival of patients who received reirradiation as part of their salvage therapy [73,74,75,76,77]. Based on these relatively small patient samples, the benefit of a second course of radiotherapy for recurrent MB appears to be greatest for relapsed, originally standard risk patients with minimal residual disease before reirradiation or no evidence of disease after surgical re-resection [73,74], those with focally recurrent disease in the brain [76], those with a response to pre-reirradiation chemotherapy [78], and patients with Group 4 tumors [75,76,77]. However, 8 out of 11 standard risk patients reported by Wetmore et al. who achieved a median survival of 5.4 years from initial diagnosis received full craniospinal reirradiation, as did a substantial proportion of patients reported by others. In addition, two of the five standard risk patients with no evidence of disease at the time of reporting received gamma knife treatment for tumor progression after reirradiation [74].

While combining radiotherapy with antiangiogenic therapy is also supported by experimental data [79,80], suggesting that the addition of radiotherapy to VEGF signaling inhibition and other targeted therapies greatly enhances the antitumor effect, we did not consider full craniospinal reirradiation given the high expected risk of late toxicities in potential long-term survivors. To compensate for the need to treat the whole CSF compartment in order to improve survival, intensive intraventricular therapy alternating etoposide and cytarabine is part of the MEMMAT strategy. That seemed all the more important since in contrast to the studies by Wetmore, where 11 out of 14 patients had standard risk disease at index diagnosis, six of our nine long-term survivors had high-risk disease at primary diagnosis (M1, *n* = 2, M2, *n* = 1, and M2/M3, *n* = 3) including one patient with a second recurrence (case 3).

The understanding of the biology of MB, which is now known to consist of a number of distinct molecular subgroups, has tremendously evolved since the MEMMAT-based antiangiogenic approach was first conceived. Regarding the impact of MB molecular group on survival, several published series [75,76,77] reported a longer median postrelapse survival primarily for patients with Group 4 tumors. This does not seem to apply to patients treated according to the MEMMAT strategy. In accordance with the COG phase II study [66] that did not show an apparent difference between MB group 3 and MB group 4, median survival has not been reached for the five patients with Group 3 tumors in our series and was 42.8 (14–71) months for the 16 patients with Group 4 tumors. Three of our nine long-term survivors had Group 3 tumors (cases 2, 8, and 29) and one patient (case 7) with an unfavorable subgroup G34_II tumor died of an accident without evidence of tumor by MRI 23 months after diagnosis of his recurrence. The only patient with a Group 3 medulloblastoma who responded neither to chemotherapy nor to antiangiogenic therapy was an infant with a subgroup G34_II tumor and a high *CMYC* amplification at primary diagnosis (case 19). Among the 14 Group 4 tumors for whom subgroup was determined, subgroup was G34_VIII in nine. Known subgroup of alive patients with Group 4 tumors was G34_VIII in three and G34 _V in two.

Regarding other molecular groups, numbers were too small to draw any conclusions with regard to response to the MEMMAT strategy. There was only one WNT and one SHH infant tumor in the series. Unfortunately, the patient with the WNT tumor was one of only two patients who did not receive intraventricular therapy. She was also not reirradiated and recurred with a combined relapse one year after enrollment in “MEMMAT-like” therapy. The infant with the SHH tumor and the biallelic MSH2 loss in her MB progressed under chemo-and antiangiogenic therapy.

Therapy was generally well-tolerated and out-patient. Toxicities were primarily hematologic, including five cases of different subtypes of AML. Except for one patient (case 16) who developed a MDS-AML with biallelic mutations of the CEBPA gene, all patients were heavily pretreated, two with conventional chemotherapy for two (case 27) and one (case 10) prior relapse, and three (cases 9, 10, and 13) were retreated with “MEMMAT-like” therapy for consecutive relapses. Two of these patients (cases 9 and 13) are alive and in remission of their leukemia and medulloblastoma. Two patients (cases 16 and 27) were not in remission of their medulloblastoma and thus not eligible for bone marrow transplant, and one patient died in remission of his AML-MLL leukemia during bone marrow transplant.

## 5. Conclusions

In conclusion, antiangiogenic therapy according to the MEMMAT strategy increased median OS and PFS of patients with recurrent medulloblastoma as compared to previous reports and may lead to long-term survival in a proportion of the patients. In contrast to most previous reports, our study did not show an inferior postrelapse survival for patients with MB Group 3 tumors versus MB Group 4. Adherence to the protocol, including intraventricular therapy, appears important, and the addition of focal radiotherapy may contribute to preventing late relapses. Further research is necessary to determine a specific molecular signature in the respective MB groups that conveys sensitivity to this particular antiangiogenic metronomic approach and may inform future modifications.

## Figures and Tables

**Table 1:** Clinical characteristics, molecular tumor profile, and outcome of patients with medulloblastoma recurrence.

Case	Age at Primary Diagnosis (Years)/ Gender	Morphology	MB Group	Subgroup	Prior Therapy (RT/CT/ HDCT)	Stage at Primary Diagnosis	No. of Recurrences	Time to Relapse from Primary Diagnosis or Prior Relapse (Months)	Type of Recurrence	Age at MEMMAT Start (Years)	Duration of MEMMAT (Months)	i.th Therapy/ VP-Shunt	Best Response	RT during MEMMAT	Status/ Follow-Up in Months after First MEMMAT Start	Duration of Follow-Up after Discontinuation of Last MEMMAT
**1°**	**12/M**	**classic**	**4**	**G34_VIII**	**+/+/−**	**M2/M3**	**1**	**132**	**M2/M3**	**24**	14 + 2 *	**Yes/no**	**CR**	**focal**	**CCR, 164+**	**149+**
**2°**	**4/F**	**classic**	**3**	**G34_IV**	**+/+/−**	**M1**	**1**	**34**	**M3**	**7**	12 + 16 *	**Yes/yes**	**CR**	**focal**	**CCR, 160+**	**132+**
**3°**	**10/M**	**classic**	**4**	**G34_VIII**	+ */+/−	**M1**	**2**	**8**	**M1**	**14**	12 + 24 *	**Yes/no**	**CR**	**no**	**CCR, 160+**	**124+**
4°	9/M	classic	NA		+ */+/−	M0	3	9	M2/M3	15	21 + 17 **	Yes/no	PR	no	DOD, 63	
**5**	**7/M**	**classic**	**Non WNT/** **non SHH**		**+/+/−**	**M0**	**1**	**69**	**Local/M3**	**13**	**19 + 12**	**Yes/yes**	**CR**	**Focal #**	**AWD,131+**	**65+**
6°	9/F	classic	WNT	WNT	+/+/−	M0	1	58	Local	14	25	No/yes	NE	no	DOD, 27	
7°	12/M	LCA	3	G34_II	+/+/−	M0	1	32	Local	15	10 + 13 *	Yes/IIIrd	CR	no	DOC, 23	
**8**	**1/F**	**classic**	**3**	**G34_IV**	**−/+/+**	**M2/M3**	**1**	**24**	**M2**	**4**	**9**	**Yes/no**	**CR**	**18Gy CSI+focal**	**CCR, 96+**	**87+**
**9**	**12/M**	**classic**	3–4 ***	G34_V ***	**+/+/−**	**M2**	**1**	**36**	**M2**	**14**	22 + 24 *	**Yes/no**	**CR**	**focal**	**CR, 134+**	**57+**
10	5/M	classic	4	G34_VIII	+/+/−	M0-1	2	3	M2/M3	8	20 + 12 *	Yes/no	CR	focal	DOC, 54	
11	4/M	classic	4	G34_V	+/+/−	M0-1	1	26	M2/M3	7	29	Yes/no	PR	focal	DOD, 44	
12	7.5/M	classic	4	G34_VIII	+/+/−	NA	1	22	M2/M3	10	24	Yes/yes	PR	no	DOD, 32	
**13**	**7/M**	**classic**	**4**	**G34_VIII**	**+/+/−**	**M2/M3**	**1**	**25**	**M1,M2**	**9**	**34**	**Yes/IIIrd**	**PR**	**focal**	**CR, 86+**	**31+**
14°	6/M	LCA	NA		+ §/+/−	M1	2	21	Local	10	6	No/yes	PR	no	DOD, 10	
15	8/M	desmoplastic	NA		+/+/−	NA	2	21	Local/M3	10	5	Yes/yes	SD	no	DOD, 6	
16	7/M	classic	4	G34_VIII	+/−/−	NA	1	10	M1–M3	8	10	Yes/yes	PR	focal	DOC, 46	
17	9/F	classic	4	G34_VIII	+/+/−	M0	1	14	M1–M3	11	12	Yes/yes	SD	focal	DOD, 26	
18	0.4/F	desmoplastic	2, SHH inf	SHH_Inf_1	−/+/−	M0	1	7	M2	1	3	Yes/no	PD	no	DOD, 5	
19	1/M	LCA	3, high *MYC* ampl	G34_II	−/+/−	M2/M3	1	4	M2/M3	1	2	Yes/yes SD	PD	no	DOD, 3	
20	10/M	classic	4	G34_VIII	+ §/+/−	M0	3	12	M2/M3	11	3	Yes/no	SD	no	DOC, 3	
21	5/M	classic	Non WNT/non SHH		+/+/−	M0	2	16	M2/M3			Yes/IIIrd	SD	focal	DOD,30	
22	4.5/F	classic	4		+ §/+/+	M3	2	18	M2/M3	10	36	Yes/no	PR	focal&	DOD,42	
**23**	**5/M**	**classic**	**4**	**G34_V**	**+/+/−**	**M0**	**1**	**26**	**Local/M2**	**7**	14 + 14 *	**Yes**	**CR**	**focal&**	**CCR, 111+**	**83+**
24	7.5/M	classic	4	G34_V	+/+/−	M0	1	32	M1–M3	10	15	Yes	CR	focal&	DOD,26	
25	6.5/F	classic	4	G34_VI	+/+/−	M0	1	12	M1–M3	7	3	Yes	PD	no	DOD,3	
26	8/M	classic	NA		+/+/−	M0	1	109	local	17	10	Yes	PD	focal	DOD,13	
27	12/M	classic	4	G34_VIII	+ §/+/−	NA	3	31	M2	17	19	Yes	PR	no	DOC,20	
28	8/F	classic	4		+/+/−	M0	1	15	M1-M3	9	8	Yes	SD	no	DOD,13	
**29**	**12/F**	**classic**	**3**		**+/+/−**	**M0**	**1**	**18**	**M3**	**14**	**11**	**Yes**	**CR**	**no**	**AWD, 97+**	**88+**

Patients alive are depicted in bold and highlighted. Abbreviations: RT, radiotherapy; CT, chemotherapy; HDCT, high-dose chemotherapy; i.th, intrathecal; VP-shunt, ventriculoperitoneal shunt; LCA, large cell anaplastic; NA, not available; NE, not evaluable (resection); CR, complete response; PR, partial response; SD, stable disease; PD, progressive disease; CCR, continuous complete remission; DOD, died of disease; DOC, died of other cause; AWD, alive with disease; °, patients included in a prior publication [43]; §, reirradiation and Gamma knife; #, second relapse; *, no oral etoposide and cyclophophamide; **, bevacizumab only; ***, at recurrence; +, ongoing.

## Data Availability

The data presented in this study are available in the article and the supplementary material.

## Supplementary Materials

The following supporting information can be downloaded at: https://www.mdpi.com/article/10.3390/cancers14205128/s1.
