# Supplementary material for: Improved Long-Term Survival of Patients with Recurrent Medulloblastoma Treated with a “MEMMAT-like” Metronomic Antiangiogenic Approach
Source: Cancers (Basel). 2022 Oct 19;14(20):5128. doi: 10.3390/cancers14205128 (PMC9601092; doi:10.3390/cancers14205128)
Supplement: Supplementary file 1 [file cancers-14-05128-s001.zip › cancers-1956355-supplementary.pdf]

Supplementary Materials:

Clinical details of selected patient groups

Patients in continuous remission after single "MEMMAT- like" therapy

Case 1 presented with a temporal metastasis and thoracolumbar leptomeningeal coating 11 years after diagnosis of a primary massively metastasized MB. He was operated on his temporal metastasis, started "MEMMAT-like" treatment and received additional radiotherapy to his spinal metastases 6.5 months after "MEMMAT-like" start. He remains in remission for 13.6 years after his recurrence. Case 2 developed a sacral dural sac metastasis 38 months after primary diagnosis, started "MEMMAT-like" therapy, received additional focal radiotherapy and is in remission for 13.3 years. Case 3 developed a second recurrence with tumor cells in the CSF eight months after a first recurrence and gamma knife treatment of a supratentorial ventricular metastasis. He received no additional radiotherapy and is in remission for 13.3 years. Case 8 presented with multiple metastases at primary diagnosis, was treated with intensive chemotherapy including HDCT but did not receive radiotherapy because of her young age. She recurred with two metastases 24 months after primary diagnosis, was started on "MEMMAT-like" and received 18 Gy craniospinal irradiation plus a boost to the two metastases before stopping MEMMAT like therapy. She remains in remission for 8 years after her recurrence. Case 23 recurred 28 months after primary diagnosis with a combined local and intraventricular relapse (right frontal horn metastasis), received gamma knife radiosurgery and "MEMMAT- like" treatment and is in remission 9.2 years after his relapse.

Patients in remission after intercurrent relapses

Two patients (cases 9, and 13) are again in remission after intercurrent relapses and retreatment with "MEMMAT like" therapy for 134 and 86 months after first starting "MEMMAT-like" therapy. Case 9 developed a right frontal metastasis three years after primary diagnosis of a metastasized medulloblastoma and received "MEMMAT like" followed by focal radiotherapy. Three years after his first recurrence the patient developed a left frontal parafalxial metastasis, that was completely resected, retreated with "MEMMAT-like" for six months followed by radiotherapy. Thirty months later the patient experienced another recurrence at the same site, was reoperated and restarted on "MEMMAT-like". Treatment had to be modified, bevacizumab and celecoxib were stopped after 3 months because of proteinuria and all treatment discontinued after 6 months. Following his third recurrence the patient was diagnosed with a promyelocytic leukemia (PML) that was treated appropriately. After discontinuation of leukemia treatment, he received another surgery for his frontal metastasis followed by daily oral everolimus as maintenance treatment for two years. As of July 2022, the patient is in good clinical condition and in remission of his leukemia and MB for 41 months and alive for 14.2 years after his primary diagnosis. Case 13 was diagnosed with two frontal metastases and tumor cells in the CSF 25 months after primary diagnosis of a massively disseminated MB. He received "MEMMAT-like" treatment followed by focal irradiation of the metastases 11 months after start of "MEMMAT-like" treatment. Twenty-eight months

after his first recurrence the patient developed another recurrence at the same prior irradiated site. The metastasis was resected and the patient restarted on "MEMMAT- like" treatment. Twenty-five months after his second recurrence he developed a third recurrence adjacent to the prior resection that was again operated on. Five months into his third "MEMMAT- like" treatment the patient developed a secondary AML (t16;21). Concomitant to the AML treatment, medulloblastoma treatment was continued with intrathecal therapy only for 9 months. Currently the patient is in remission of his leukemia and his MB 37 months after his third recurrence and 8.4 years after his original diagnosis.

Patients alive with late recurrences

Case 5 presented with a MB recurrence in the posterior fossa and spinal canal with metastases L2-L3 and L5-S1, 5.5 years after primary diagnosis. After partial resection of the tumor in the posterior fossa and a few courses of chemotherapy to bridge the time to "MEMMAT-like" start the patient received "MEMMAT-like" therapy for two years (second year without etoposide or cyclophosphamide). No additional radiotherapy was performed. Five years after his first combined local and metastatic recurrence the patient experienced a second recurrence at the site of the upper spinal metastasis with tumor cells in the CSF. He received full "MEMMAT- like" therapy for seven months with concomitant focal radiotherapy after six months. He again remained in remission for almost five years when he developed a local recurrence in the posterior fossa, was reoperated and is currently receiving TMZ.

Case 29 recurred with a thoracic spinal metastasis 18 months after primary diagnosis, received MEMMAT like treatment without focal radiotherapy for almost one year, when all medication was stopped because of side effects (hypertension, reduced kidney function and restrictive pulmonary disease). The patient recovered from her side effects except for a residual decreased pulmonary function, remained in remission for almost six years when her spinal metastasis slowly recurred and was treated with local radiotherapy only. Thirteen months later new spinal metastases appeared outside of the irradiation field and the patient was started on TMZ.

Selected patients of interest

Case 4, our index patient, recurred three years after primary diagnosis of a standard risk MB, achieved a second remission following double high-dose chemotherapy and reirradiation of his multiple cerebral and spinal metastases. He recurred again one and a half years later and was among the first patients to be treated on the "5-drug" trial [41,42]. He achieved an almost complete response after six months when treatment was discontinued but developed a third recurrence only three months later with metastases in the third and fourth ventricle. Because of platelet counts hovering between 30.000 and 40.000 treatment with the "5-drug" regimen was resumed at a reduced dose of etoposide and cyclophosphamide and intrathecal therapy consisting of alternating etoposide and liposomal cytarabine as well as intravenous bevacizumab every two weeks were added. The patient achieved a partial remission despite intermittent breaks and oral etoposide and cyclophosphamide had to be discontinued completely after 2.2 years. Upon increase of the residual tumors five months later bevacizumab was reinitiated as monotherapy for 17 months also

leading to a minor response over one year as confirmed by MRI. The patient eventually died of tumor progression five years and three months after his third recurrence and enrollment in “MEMMAT like” therapy.

Case 7 presented with a locally recurrent WNT pathway tumor 4.8 years after primary diagnosis. Her tumor was resected and she received MEMMAT like albeit without intrathecal therapy because of a VP-shunt in place. She recurred one year later, was reirradiated but progressed locally and with leptomeningeal metastases and succumbed to her disease.

Case 18, an infant with Lynch syndrome, developed a recurrence of her SHH infant tumor while under chemotherapy seven months after primary diagnosis. She had lost the second mismatch repair *MSH2* allele in the tumor and did not respond to ”MEMMAT-like” therapy. Similarly, case 19, an infant with a high *CMYC* amplified Group 3 tumor did not respond to therapy.

Patients who died of another cause

Case 7 died of an accident in complete remission 23 months after his recurrence. Case 10 died in remission of his brain tumor of septicemia during BMT for leukemia. Case 16 and 27 were not eligible for BMT because of residual brain tumor and died of leukemia and case 20 died of a septic shock.
